# Supplementary material for: Association of smoking status with hospitalisation for COVID-19 compared with other respiratory viruses a year previous: a case-control study at a single UK National Health Service trust
Source: F1000Res. 2022 Jan 17;10:846. Originally published 2021 Aug 24. [Version 3] doi: 10.12688/f1000research.55502.3 (PMC8796008; doi:10.12688/f1000research.55502.3)
Supplement: Peer review history

# Peer review: Reviewer response for version 3

**DOI:** 10.5256/f1000research.120034.r120026

**Published:** 2022-01-27

**Authors:** Jonathan M. Samet

**Affiliations:** 1 Colorado School of Public Health, Aurora, CO, USA

My two remaining concerns have been satisfactorily addressed and the manuscript is suitable for indexing.

Is the work clearly and accurately presented and does it cite the current literature?

Partly

If applicable, is the statistical analysis and its interpretation appropriate?

Yes

Are all the source data underlying the results available to ensure full reproducibility?

Partly

Is the study design appropriate and is the work technically sound?

Partly

Are the conclusions drawn adequately supported by the results?

No

Are sufficient details of methods and analysis provided to allow replication by others?

Partly

Reviewer Expertise:

Pulmonary disease, epidemiology, tobacco control

I confirm that I have read this submission and believe that I have an appropriate level of expertise to confirm that it is of an acceptable scientific standard.

# Peer review: Reviewer response for version 2

**DOI:** 10.5256/f1000research.79264.r100232

**Published:** 2021-11-25

**Authors:** Maria Rosaria Galanti

**Affiliations:** 1 Department of Global Public Health, Karolinska Institutet, Stockholm, Sweden

The authors did a considerable effort to comply with the review, and the article is now acceptable for indexing.

Is the work clearly and accurately presented and does it cite the current literature?

Yes

If applicable, is the statistical analysis and its interpretation appropriate?

Yes

Are all the source data underlying the results available to ensure full reproducibility?

Yes

Is the study design appropriate and is the work technically sound?

Partly

Are the conclusions drawn adequately supported by the results?

Partly

Are sufficient details of methods and analysis provided to allow replication by others?

Yes

Reviewer Expertise:

Epidemiology, tobacco use

I confirm that I have read this submission and believe that I have an appropriate level of expertise to confirm that it is of an acceptable scientific standard.

# Peer review: Reviewer response for version 2

**DOI:** 10.5256/f1000research.79264.r100233

**Published:** 2021-11-16

**Authors:** Jonathan M. Samet

**Affiliations:** 1 Colorado School of Public Health, Aurora, CO, USA

1. With regard to the exposure classification, in the instances when the notes and the HER were in disagreement, how was exposure classified?  There does not seem to be a specification as to how this situation was handled.
2. Smoking has been causally associated with respiratory infections, including influenza (see the 2014 report of the US Surgeon General). Consider the implications for interpretation of the findings. 

Is the work clearly and accurately presented and does it cite the current literature?

Partly

If applicable, is the statistical analysis and its interpretation appropriate?

Yes

Are all the source data underlying the results available to ensure full reproducibility?

Partly

Is the study design appropriate and is the work technically sound?

Partly

Are the conclusions drawn adequately supported by the results?

No

Are sufficient details of methods and analysis provided to allow replication by others?

Partly

Reviewer Expertise:

Pulmonary disease, epidemiology, tobacco control

I confirm that I have read this submission and believe that I have an appropriate level of expertise to confirm that it is of an acceptable scientific standard, however I have significant reservations, as outlined above.

# Response

**Published:** 2022-01-05

**Authors:** Olga Perski

**Affiliations:** University College London, UK

Thank you for your helpful comments.

**With regard to the exposure classification, in the instances when the notes and the HER were in disagreement, how was exposure classified?  There does not seem to be a specification as to how this situation was handled.**

 We have now clarified this in the Methods section (within the ‘Exposure variable’ subsection):

 “The most recent record of smoking status, obtained from either the summary EHR or the contemporaneous medical notes, was extracted. Where there was discordance between the summary EHR and the contemporaneous medical notes, smoking status was classified based on the contemporaneous medical notes (as this clinician obtained information was assumed to be more recent and therefore accurate).“

**Smoking has been causally associated with respiratory infections, including influenza (see the 2014 report of the US Surgeon General). Consider the implications for interpretation of the findings.**

 Thank you – we now refer to the US Surgeon General’s report in the Discussion:

 “As concluded in the US Surgeon General’s 2014 report, smoking and infection with acute respiratory illnesses (including pneumonia) appear causally related. However, evidence as to the association between smoking and increased disease severity (once infected) is currently lacking.”

# Peer review: Reviewer response for version 1

**DOI:** 10.5256/f1000research.59083.r94125

**Published:** 2021-10-08

**Authors:** Jonathan M. Samet

**Affiliations:** 1 Colorado School of Public Health, Aurora, CO, USA

**General Comments:**

 This paper describes the findings of a hospital-based case-control study on cigarette smoking on risk for hospitalization from COVID-19. The paper is lacking a clear explication of the underlying hypothesis, which is critical to determining what is the appropriate control group. Smoking might influence the risk of becoming infected with SARS-CoV-2; and/or the risk of more severe disease given infection, which could lead to increased risk for hospitalization and death. The effects of smoking on airway receptors could plausibly effect risk for infection and modify its severity, as could the effects of smoking on the lungs and its causation of non-communicable diseases. 

 The authors write extensively about the problems of control group selection but, lacking a clearly focused hypothesis, their discussion is general. If the question is whether smoking affects the risk of hospitalization, given infection, then the appropriate control group comprises non-hospitalized, SARS-CoV-2 infection who were not hospitalized. If the question related to risk for infection, than the appropriate control group has comparable exposure but without becoming infected. 

 The authors turn to a problematic historical control group of individuals hospitalized with other respiratory infections during a pre-pandemic time period. How this control group relates to the potential hypotheses is unclear. Additionally, there may have been pandemic-related trends in smoking that would not be taken into account. Additionally, smoking increases risk for respiratory infections generally (see 2014 report of the US Surgeon General ^1^), further complicating interpretation of the results. 

 Why is SEP included in the model? It is a powerful predictor of smoking and of co-morbidities, but is it a likely confounder and why? 

 Were the cases all diagnosed with COVID-19? What about those admitted for other reasons, but testing positive for SARS-CoV-2 on admission?

 The discussion does not adequately integrate this new study into existing literature, including ongoing systematic reviews.

Is the work clearly and accurately presented and does it cite the current literature?

Partly

If applicable, is the statistical analysis and its interpretation appropriate?

Yes

Are all the source data underlying the results available to ensure full reproducibility?

Partly

Is the study design appropriate and is the work technically sound?

Partly

Are the conclusions drawn adequately supported by the results?

No

Are sufficient details of methods and analysis provided to allow replication by others?

Partly

Reviewer Expertise:

Pulmonary disease, epidemiology, tobacco control

I confirm that I have read this submission and believe that I have an appropriate level of expertise to state that I do not consider it to be of an acceptable scientific standard, for reasons outlined above.

## References

[1] : The Health Consequences of Smoking—50 Years of Progress: A Report of the Surgeon General.2014; 24455788

# Response

**Published:** 2021-11-08

**Authors:** Olga Perski

**Affiliations:** University College London, UK

***This paper describes the findings of a hospital-based case-control study on cigarette smoking on risk for hospitalization from COVID-19. The paper is lacking a clear explication of the underlying hypothesis, which is critical to determining what is the appropriate control group. Smoking might influence the risk of becoming infected with SARS-CoV-2; and/or the risk of more severe disease given infection, which could lead to increased risk for hospitalization and death. The effects of smoking on airway receptors could plausibly effect risk for infection and modify its severity, as could the effects of smoking on the lungs and its causation of non-communicable diseases.***

***The authors write extensively about the problems of control group selection but, lacking a clearly focused hypothesis, their discussion is general. If the question is whether smoking affects the risk of hospitalization, given infection, then the appropriate control group comprises non-hospitalized, SARS-CoV-2 infection who were not hospitalized. If the question related to risk for infection, than the appropriate control group has comparable exposure but without becoming infected.***

***The authors turn to a problematic historical control group of individuals hospitalized with other respiratory infections during a pre-pandemic time period. How this control group relates to the potential hypotheses is unclear.***

 Thank you for the constructive feedback on our paper, which we believe have helped strengthen it.

 We agree that the selection of controls ultimately mixes two potential mechanisms through which smoking status may influence COVID-19 outcomes (i.e., comparable exposure leading to differential infection rates and/or comparable infection rates leading to differential hospitalisation rates). As elaborated on in the discussion, and now clarified in the introduction on p.4-5, we selected the controls on the basis of sharing a similar route of transmission (i.e., influenza) and risk factors for hospitalisation as cases. Since planning this study in early 2020, the research community has learnt more about COVID-19 infection and disease mechanisms, and we have ourselves taken more care to map out potential causal pathways that need to be considered when planning studies to examine the relationship of smoking status and COVID-19 infection/disease to avoid confounding (see Perski et al., 2021). We now discuss these considerations and the issues with the selection of the controls on p.13-14:

 “Finally, we have argued elsewhere that studies that aim to elucidate the potential causal relationship between smoking status and COVID-19 should first clarify the mechanism(s) through which smoking and/or nicotine use is expected to influence COVID-19 infection and/or disease outcomes contingent on infection and plan their studies accordingly (Perski et al., 2021). Considering these reflections, and following constructive peer review, we acknowledge that the present study through its selection of the control sample does not distinguish between two potential mechanisms: comparable exposure to SARS-CoV-2 virus with differential infection in current compared with never smokers, and comparable SARS-CoV-2 infection with differential hospitalisation in current compared with never smokers. Future research should aim to isolate these potential mechanisms by design, thus getting closer to unbiased estimates of the association of smoking/nicotine use with COVID-19 outcomes. However, we note that – given the time-varying dynamics of respiratory viruses – it would be incredibly challenging to design a representative study to compare rates of infection in those with similar exposure using available datasets. Similarly, as data linkage of representative infection surveys with hospital records is also limited at present, designing a study to examine the second potential pathway (i.e., examining hospitalisation rates in a representative sample of those infected) would also be very challenging.”

***Additionally, there may have been pandemic-related trends in smoking that would not be taken into account.***

 With regards to pandemic-related trends in smoking, we have now added the following to the discussion on p.14:

 “However, a single year was used for the selection of controls, and there was no significant change in smoking prevalence among all adults in England between immediately before and early in the pandemic (Apr-Jul 2020, Jackson et al., 2020), so we expect any potential impact of pandemic-related trends in smoking prevalence on the results to be minimal.”

 Additionally, smoking increases the risk for respiratory infections generally (see 2014 report of the US Surgeon General1), further complicating interpretation of the results.

 As the controls came from a similar population (i.e., individuals at risk of infection and hospitalisation with a circulating respiratory disease), we believe this was a strength in their selection. We have therefore not seen it necessary to discuss this beyond what is already mentioned about the selection of the control sample.

***Why is SEP included in the model? It is a powerful predictor of smoking and of co-morbidities, but is it a likely confounder, and why?***

 SEP was included as a potential confounder due to being a strong predictor of both smoking and COVID-19 hospitalisation. In response to a similar comment from Reviewer 1, we have now included a sensitivity analysis with SEP removed (reported on p.10), with results remaining similar.

***Were the cases all diagnosed with COVID-19? What about those admitted for other reasons, but testing positive for SARS-CoV-2 on admission?***

 Thank you for highlighting – all patients included in the case series had PCR confirmed infection with COVID-19. Given the broad and currently poorly understood COVID-19 pathology following infection, it was not possible to exclude patients with incident positive tests based on their presenting symptoms to ensure the exclusion of those testing positive upon admission to hospital who were admitted for reasons other than COVID-19. This is an important potential source of bias and we have now included the following in the limitations on p.13:

 “Seventh, given the broad and currently poorly understood COVID-19 pathology following SARS-CoV-2 infection, it was not possible to differentiate patients presenting to hospital due to COVID-19 or those admitted for other reasons. This may have introduced additional bias in that individuals may have been admitted to hospital with co-incident SARS-CoV-2 infection that was not associated with their current health need. We tried to mitigate this by only including cases if they had a positive COVID-19 test result less than 5 days from their date of hospital admission.”

***The discussion does not adequately integrate this new study into existing literature, including ongoing systematic reviews.***

 We have now included a paragraph in the discussion on p.11 which highlights how our findings compare with those from an ongoing systematic review:

 “A living review and unadjusted Bayesian meta-analyses of observational studies conducted during the COVID-19 pandemic, up-to-date as of July 2021, has found that current smokers appear to be at reduced risk of COVID-19 infection, but that there is inconclusive evidence of an increased risk of more severe disease among smokers who are infected. In addition, former smokers appear to be at increased risk of severe disease and mortality from COVID-19 (Simons et al., 2020; Simons et al., 2021).”

# Peer review: Reviewer response for version 1

**DOI:** 10.5256/f1000research.59083.r92725

**Published:** 2021-09-13

**Authors:** Maria Rosaria Galanti

**Affiliations:** 1 Department of Global Public Health, Karolinska Institutet, Stockholm, Sweden

This was a case-control study aiming at shedding light on the potential causal association between tobacco smoking and hospitalization because of COVID-19. In the light of the controversial associations between smoking and risk for infection with SARS-COV-2 on the one side, clinical disease and its severity on the other side, the contribution has a great interest. The manuscript is clearly and soundly written, and the results are thoughtfully discussed within a series of sensitivity analyses.

 However, the several weaknesses in the design and assessment of exposure, with corresponding risk for bias (to be sure, fully acknowledged by the authors) did not allow the attainment of the study's goal. Besides the weaknesses already addressed by the authors, the following points should be considered, bearing in mind that in a case-control study the concern is on characteristics being different or differentially assessed in the case and in the referent series and possibly associated with the exposure:

1. Can we assume that the procedure for hospital admissions for COVID-19 was the same as for other respiratory infections one year earlier (see page 6, lines 3-4)? To my knowledge, hospital admission routines during the pandemic were profoundly disrupted in many countries, with patient transfer between hospitals, different priorities of admission, etc. Even if not explicitly, some of the patient characteristics correlated with smoking may have determined different patient selection and/or ascertainment of smoking.
2. Could the assessment of smoking at the point of hospitalization among COVID-19 patients be biased by the pandemic or by the disease? Starting from March 2020, much attention was drawn on smoking, causing adverse outcomes in COVID-19 hospitalized patients, a position even endorsed by the WHO later in Spring 2020 ( https://www.who.int/news-room/commentaries/detail/smoking-and-covid-19). Thereafter, some smokers (especially if already suffering from smoking-related diseases) may have quit before hospital admission or may have concealed their smoking to the healthcare staff. Also, the accuracy of the staff assessing smoking behavior may have varied depending on the severity of the disease. This risk is supported by the notation that the COVID-19 patients with unknown smoking status (more numerous than among the referent cases) had higher mortality than those with a full assessment of smoking. Following the premises in the introduction (logical expectation of higher risk among smokers), it would be sensible to assume that among these “unknown smoking” patients the proportion of smokers would be higher. When tested in a sensitivity analysis, this assumption made the inverse association smoking-COVID-19 hospital admission weaken/disappear (page 8).
3. The adjustment for SEP was done on the ecologic level. This may have introduced bias (over-adjustment for opportunity of infection, including due to high smoking prevalence?) if the rate of infection/hospital admission with SARS-COV-2 was differently clustered in socio-economically disadvantaged areas compared to affluent areas, at odds with the more “widespread” diffusion of other respiratory infections.

Some minor points:

1. I would avoid the notation “case cohort” and “control cohort” (page 6), which is misleading, using rather “case series” or “case sample”.
2. I would rephrase the conclusions as “…patients hospitalized with COVID-19 had lower odds of appearing as smokers compared with patients... “, which is more respectful of what was actually analyzed.

Is the work clearly and accurately presented and does it cite the current literature?

Yes

If applicable, is the statistical analysis and its interpretation appropriate?

Yes

Are all the source data underlying the results available to ensure full reproducibility?

Yes

Is the study design appropriate and is the work technically sound?

Partly

Are the conclusions drawn adequately supported by the results?

Partly

Are sufficient details of methods and analysis provided to allow replication by others?

Yes

Reviewer Expertise:

Epidemiology, tobacco use

I confirm that I have read this submission and believe that I have an appropriate level of expertise to state that I do not consider it to be of an acceptable scientific standard, for reasons outlined above.

# Response

**Published:** 2021-11-08

**Authors:** Olga Perski

**Affiliations:** University College London, UK

***This was a case-control study aiming at shedding light on the potential causal association between tobacco smoking and hospitalization because of COVID-19. In the light of the controversial associations between smoking and risk for infection with SARS-COV-2 on the one side, clinical disease and its severity on the other side, the contribution has a great interest. The manuscript is clearly and soundly written, and the results are thoughtfully discussed within a series of sensitivity analyses.***

 Thank you for the constructive feedback on our manuscript, which we believe has helped strengthen it.

***However, the several weaknesses in the design and assessment of exposure, with the corresponding risk for bias (to be sure, fully acknowledged by the authors) did not allow the attainment of the study's goal. Besides the weaknesses already addressed by the authors, the following points should be considered, bearing in mind that in a case-control study the concern is on characteristics being different or differentially assessed in the case and in the referent series and possibly associated with the exposure:***

***Can we assume that the procedure for hospital admissions for COVID-19 was the same as for other respiratory infections one year earlier (see page 6, lines 3-4)? To my knowledge, hospital admission routines during the pandemic were profoundly disrupted in many countries, with patient transfer between hospitals, different priorities of admission, etc. Even if not explicitly, some of the patient characteristics correlated with smoking may have determined different patient selection and/or ascertainment of smoking.***

***Could the assessment of smoking at the point of hospitalization among COVID-19 patients be biased by the pandemic or by the disease? Starting from March 2020, much attention was drawn on smoking, causing adverse outcomes in COVID-19 hospitalized patients, a position even endorsed by the WHO later in Spring 2020 (https://www.who.int/news-room/commentaries/detail/smoking-and-covid-19). Thereafter, some smokers (especially if already suffering from smoking-related diseases) may have quit before hospital admission or may have concealed their smoking to the healthcare staff. Also, the accuracy of the staff assessing smoking behavior may have varied depending on the severity of the disease. This risk is supported by the notation that the COVID-19 patients with unknown smoking status (more numerous than among the referent cases) had higher mortality than those with a full assessment of smoking. Following the premises in the introduction (logical expectation of higher risk among smokers), it would be sensible to assume that among these “unknown smoking” patients the proportion of smokers would be higher. When tested in a sensitivity analysis, this assumption made the inverse association smoking-COVID-19 hospital admission weaken/disappear (page 8).***

 Thank you - we have now reflected on these important points in the limitations section on p.12:

 “Fifth, hospital admission routines were severely disrupted in many countries during the early stages of the COVID-19 pandemic (e.g., patient transfer between hospitals, altered admission criteria). This may have influenced patient selection and/or the recording of smoking status. With regards to patient selection, albeit possible, we are unaware of evidence from UK hospitals of patient characteristics correlated with smoking (e.g., age, SEP) determining whether a patient would be admitted, rather than their clinical situation on presentation. For example, smoking status has not been included in a widely used hospital-based algorithm for predicting clinical deterioration from COVID-19 (i.e., the ISARIC-4C deterioration score; Gupta et al., 2021). With regards to the recording of smoking status, to the best of our knowledge, the admission routines (including the medical history taking) within the selected hospital trust remained stable during the pandemic. However, patients admitted with COVID-19 may generally have been more unwell at the point of admission compared with controls, which may have led to less accurate documentation of smoking status. This potential bias was somewhat reduced by those surviving their COVID-19 illness being followed up through a specialised respiratory medicine clinic implemented during the pandemic, with smoking status ascertained during follow-up calls/visits. However, the finding that COVID-19 patients with unknown (compared with those with recorded) smoking status had greater in-hospital mortality means that residual bias cannot be ruled out. A sensitivity analysis with all missing assumed to be current smokers made the negative association of smoking status and COVID-19 hospital admission go away. However, it should be noted that this is a very strong assumption, unlikely to hold true.”

***The adjustment for SEP was done on the ecologic level. This may have introduced bias (over-adjustment for the opportunity of infection, including due to high smoking prevalence?) if the rate of infection/hospital admission with SARS-COV-2 was differently clustered in socio-economically disadvantaged areas compared to affluent areas, at odds with the more “widespread” diffusion of other respiratory infections.***

 To investigate the role of SEP as a potential confounder in our analysis, we have now conducted an additional sensitivity analysis removing SEP from the multivariable model (reported on p.10). In the sensitivity analysis with sex and age only, current smokers had reduced odds of being hospitalised with COVID-19 compared with other respiratory viruses a year previous (OR = 0.51, 95% CI = 0.31-0.86, *p* = 0.01). There was no significant association among former smokers (OR = 0.95, 95% CI = 0.65-1.40, *p *= 0.80). These were similar to the analyses including SEP (OR = 0.48, 95% CI = 0.28-0.83, p < 0.01 and OR = 0.90, 95% CI = 0.61-1.34, p = 0.61, respectively).

***Some minor points:***

***I would avoid the notation “case-cohort” and “control cohort” (page 6), which is misleading, using rather “case series” or “case sample”.***

 Thank you for pointing out this difference – we have now changed to “case sample” throughout.

***I would rephrase the conclusions as “…patients hospitalized with COVID-19 had lower odds of appearing as smokers compared with patients... “, which is more respectful of what was actually analyzed.***

 This has now been amended accordingly.
